# Heavy metal pollution and environmental risks in the water of Rongna River caused by natural AMD around Tiegelongnan copper deposit, Northern Tibet, China

**DOI:** 10.1371/journal.pone.0266700

**Published:** 2022-04-07

**Authors:** Yuhu Luo, Jiaoping Rao, Qinxian Jia

**Affiliations:** 1 Institute of Land Engineering & Technology, Shaanxi Provincial Land Engineering Construction Group Co., Ltd., Xi’an, China; 2 Shaanxi Provincial Land Engineering Construction Group Co., Ltd., Xi’an, China; 3 Key Laboratory of Degraded and Unused Land Consolidation Engineering, the Ministry of Natural Resources, Xi’an, China; 4 Shaanxi Provincial Land Consolidation Engineering Technology Research Center, Xi’an, China; 5 China University of Geosciences (Beijing), Beijing, China; 6 Key Laboratory of Metallageny and Mineral Assessment, Ministry of Natural resources, Institute of Mineral Resources, Chinese Academy of Geological Sciences, Beijing, China; 7 Key Laboratory of Saline Lake Resources and Environments, Ministry of Natural resources, Institute of Mineral resources, Chinese Academy of Geological Sciences, Beijing, China; Tsinghua University, CHINA

## Abstract

Acid mine drainage (AMD) is one of the biggest environmental challenges associated with in the mining process. Most of the current research on AMD focuses on developed deposits, whereas there is almost no research on naturally-produced AMD from undeveloped deposits. In this study, river water and AMD were collected to analyze the distribution characteristics of heavy metals and the phytoplankton community. In addition, the environmental risks of heavy metals were evaluated by single-factor pollution index, Nemerow pollution index and health risk assessment model. The results show that the pH of the Rongna River water ranged from 6.52 to 8.46, and the average concentrations of Mn and Ni were 867.37 and 28.44 μg/L, respectively, which exceed the corresponding Grade III Environmental Quality Standard of Surface Water. The results of the environmental health risk assessment show that the river section of the Rongna River was seriously polluted by the heavy metal Mn after AMD confluence, and the health risk assessment indicates that oral ingestion of Mn posed a potential non-carcinogenic risk to children and adults. A total of 35 phytoplankton species were found in the Rongna River. The phytoplankton biomass was negatively correlated with the concentration of major heavy metals, indicating that the heavy metal concentration exceeded the tolerance limit of phytoplankton, thereby affecting their normal growth. Finally, statistical analysis shows that Cu, Zn, Ni, Mn and Cd in the Rongna River were mainly derived from AMD.

## Introduction

The exploitation of mineral resources provides human beings with a large amount of resources and energy, but also causes heavy metal pollution in water [1, 2]. Previous studies have shown that rivers flowing through mining areas are more susceptible to heavy metal pollution [3, 4]. Due to both man-made and natural factors, some deposits rich in sulfides (pyrite, chalcopyrite, galena, etc.) are exposed to air, and through the action of extended periods of rain and weathering, a large amount of acid mine drainage (AMD) rich in heavy metal ions is formed [5, 6]. When this AMD enters a river, it causes great harm to the ecological environment of the given water body [7, 8]. After entering the environment, some toxic heavy metals are not only non-biodegradable, but they also accumulate in the environment [9]. Furthermore, some dissolved heavy metals are very easily used by aquatic organisms [10], and may also enter the human body through drinking water, skin absorption and biological chains, ultimately endangering human health [11, 12].

Remediating heavy metal pollution of rivers caused by mineral mining is often costly, time-consuming and labor-intensive, and immediate results are difficult to achieve [13]. Even many years after mining has ceased, the impact of heavy metals on the environment still exists [14, 15]. For example, although the abandoned lead-zinc mine in northern Idaho in the western United States has been closed for 75 years, it still has a significant impact on the river ecosystem of the region [16]. Moreover, acidic wastewater enhances the solubility of heavy metals, which allows them to migrate long distances, thereby causing harm to rivers, nearby soil and even groundwater [17]. Operations at China’s Dexing copper mine have caused serious river pollution around the mining area, which has spread to farmland soil through the use of river water for irrigation [18]. China’s Dabaoshan iron polymetallic mine has formed a large amount of tailings and accumulated substantial waste rock, which are quickly oxidized after being in contact with air, resulting in acidic wastewater. At the same time, a large amount of toxic and harmful heavy metal ions are released, causing serious pollution of the Hengshi River within the mining area. The pollution has spread to the downstream town of Xinjiang, causing the death of a large number of fish and shrimp in the river [19]. The highly toxic, large-scale pollution caused by the acid wastewater from the mine has caused devastating harm to the ecological environment of the area.

**Fig 1:**
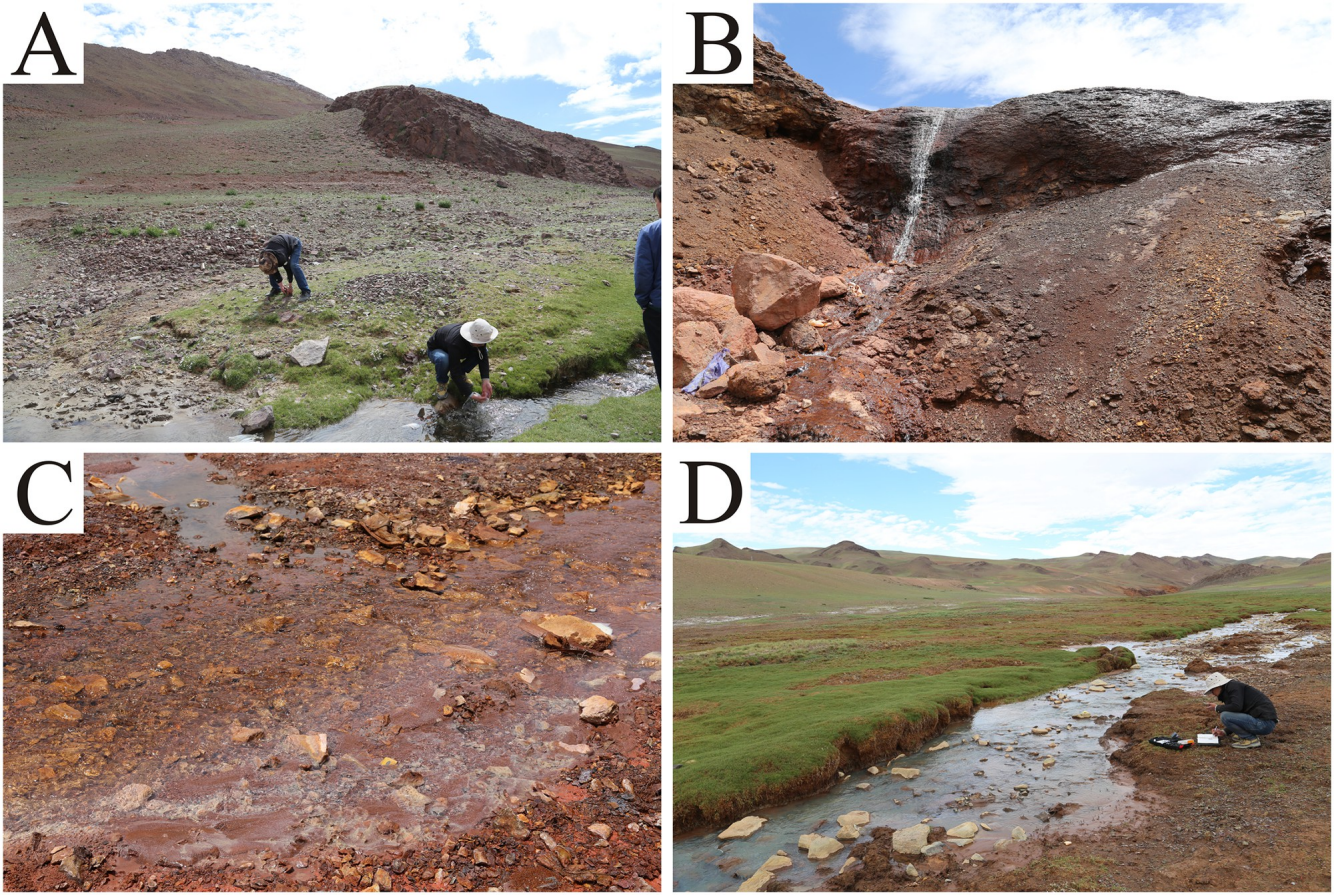
Photographs of the Rongna River. Panel A represents the uncontaminated upstream section of the Rongna River; panel B represents the AMD from the deposit; panel C represents the river section after the AMD flows into it; and panel D represents the river section far away from the deposit area.

The Tiegelongnan copper deposit is located in the hinterland of the northern Tibetan Plateau, which represents a fragile ecological environment. The copper mine belongs to a super-large high-sulfur, porphyry-type epithermal copper deposit with a preliminary estimated copper ore reserve of more than 11 million tons [20]. At present, the deposit has not been mined, but part of the ore body is exposed to air and easily oxidizes to form acidic wastewater. The Rongna River, originating from a mountain spring far away from the Tiegelongnan copper deposit, is about 30 kilometers long. When the Rongna River flows through the Tiegelongnan copper deposit, AMD (Fig 1B) that is naturally formed in the middle of the ore body flows into the river. As a result, a large amount of mineral extracts are carried into the Rongna River, causing serious harm to the river’s ecological environment. It can be seen from field observations that before pollution from the deposit flows in, the vegetation on both sides of the river bank is luxuriant (Fig 1A). However, the vegetation disappears from areas located after the AMD flows into the river (Fig 1C) and many yellow bubbles appear in the water, indicating that the influx of AMD causes serious water pollution in the Rongna River. In this study, the concentration of heavy metals, pH, and phytoplankton distribution characteristics in river water were determined to assess environmental risk by single-factor pollution index, Nemerow pollution index and human health risk assessment model. The purpose of this study was to (1) investigate the concentrations, spatial distributions and sources of Cu, Pb, Zn, As, Mn, Cd, Cr, Ni, and Hg in the water of the Rongna River; (2) evaluate the environmental risk of heavy metal pollution in the river water, and evaluate the carcinogenic and non-carcinogenic risks caused by heavy metals; and (3) analyze the influence of heavy metal pollution on the distribution characteristics of phytoplankton. In view of the distinct ecological environment in northern Tibetan and the existence of naturally occurring AMD, the results of this study can provide a reference for investigating heavy metal pollution in rivers under special geographical environment and conditions.

## Materials and methods

### Study area

The Tiegelongnan copper deposit is located in the Wuma Township, Gaize County, Northern Tibet. It lies between 83°23’ E-83°27’ E longitude and 32°47’ N-32°50′ N latitude, at an altitude of 4800–5100 m. The study area belongs to a plateau subtropical semi-arid monsoon climate. The annual average temperature is -0.1°C to -2.5°C, with a large temperature difference between day and night. The annual rainfall is 308.3 mm, and the rainy season is concentrated from July to August. The Tiegelongnan copper deposit is a large-scale polymetallic sulfide deposit with an average Cu grade of 0.64% with copper resources exceeding 11 million tons. The metal minerals within the deposit include chalcopyrite, pyrite, bornite, magnetite, iron ore, sphalerite, blue chalcocite and malachite [20].

### Experimental reagents and characterization of materials

Suprapur nitric acid, 4% formaldehyde solution, Lugol’s solution, standard solution from Center of National Standard Reference Material of China (GSB04-17672004), 0.45μm glass fiber filter membrane, No. 25 plankton net (200 mesh), polyethylene sampling bottle, portable multi-parametric meter (HI9828 HANNA Italy).

### Sample collection and chemical analysis

**Fig 2:**
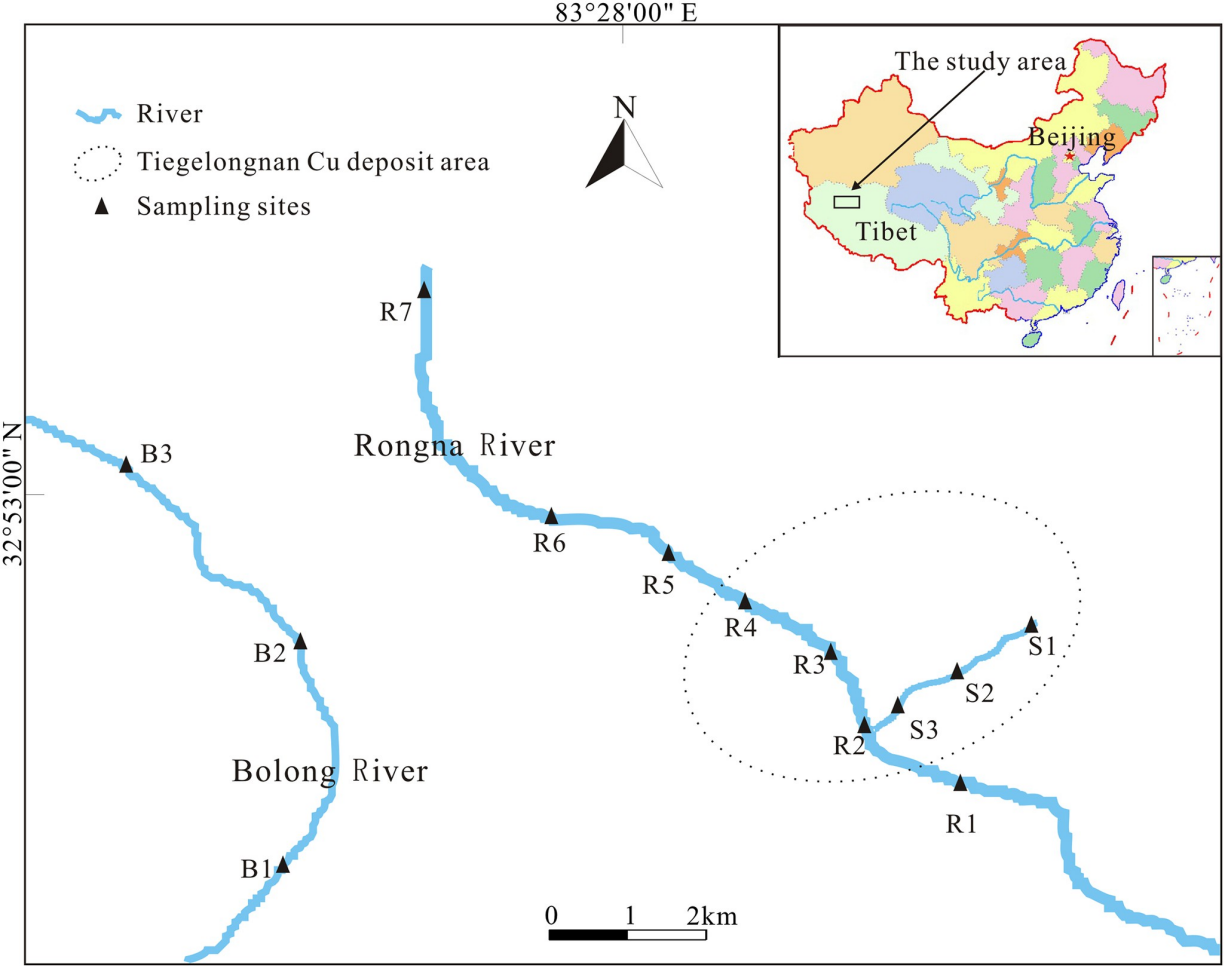
Location of the study area and the distribution of sampling points. The location coordinate map of the study area was drawn according to the USGS National Map Viewer.

Seven sampling points (R1-R7) were chosen from the uncontaminated upper reaches of the Rongna River to the end of the river, and three sampling points (S1-S3) were set up in the AMD section. At the same time, water samples from the Bolong River (BL1-BL3), away from the mining area, were collected as a control (Fig 2). Water samples for heavy metals and phytoplankton analysis were collected at each point, and a portable multi-parameter meter (HI9828, HANNA, Italy) was used to determine the pH of the water on site.

The samples used for heavy metal determination were filtered through 0.45 μm glass fiber filter membranes immediately after collection to remove large suspended solids. Next, HNO_3_ was added to ensure the pH was less than 2, and then the samples were kept sealed at 4°C. After being transported to the laboratory, Cu, Pb, Zn, Mn, Cd, Cr and Ni in the water samples were measured by inductively coupled plasma mass spectrometer system (ICP-MAS, PE300D), while As and Hg were measured by atomic fluorescence spectrometry (AFS-9760). The precision and accuracy for the analysis of heavy metals in water were validated using standard reference materials from the Center of National Standard Reference Material of China (GSB04-1767-2004). The recovery rates of heavy metal contents in the standard reference materials were between 90% and 110%. For quantitative analysis of phytoplankton, No. 25 plankton net was used to collect samples under the water surface. The collected phytoplankton-containing water samples were allowed to stand for 24 hours, and then the supernatant was carefully drawn with a pipette and concentrated to 50 mL, and 4% formaldehyde was added as a fixative. For quantification of phytoplankton, 1 L of water was collected at each sampling point, and 10 mL of Lugol’s solution was added for fixation. The water samples were returned to the laboratory and then concentrated to 100 mL. Species identification and cell counts were performed under a microscope (Olympus CX21) at 400 times magnification [21, 22].

### Environmental risk assessment

#### Pollution index

The single-factor pollution index and Nemerow pollution index are often used to evaluate the pollution status of heavy metals in water bodies [23, 24]. The single-factor evaluation method, which evaluates the pollution level of a single heavy metal in river water, was calculated as shown in Eq (1) [25]:

$${\text{P}}_{\text{i}}{=\text{C}}_{\text{i}}{\text{/B}}_{\text{i}}$$ (1)

where, P_i_ represents the single-factor pollution index of element i; C_i_ represents the actual concentration of element i (μg/L); and B_i_ represents the evaluation standard of element i. In this study, the surface water environmental quality standard of the National Environmental Protection Agency of China (NEPAC) was used as the evaluation standard (GB 3838–2002) [26].

The Nemerow pollution index not only reflects the pollution degree of single-factor heavy metals, but also describes the comprehensive pollution of multiple heavy metals. Additionally, it highlights the impact and effect of the pollutant with the largest pollution index on environmental quality. This index is currently used most frequently because it is a comprehensive method that evaluates the environmental quality of water bodies. The Nemerow pollution index was calculated as shown in Eq (2) [27].

$${\text{P}}_{\text{n}}=\sqrt{\text{0}.5\times [\text{max}{\left({\text{P}}_{\text{i}}\right)}^{\text{2}}{\text{+ave(P}}_{\text{i}}{\text{)}}^{\text{2}}\text{]}}$$ (2)

where, C_i_ represents the measured concentration of heavy metal i; max(P_i_) represents the maximum value of the heavy metal single-factor pollution index; and ave(P_i_) represents the average value of the single-factor pollution index for each heavy metal. The pollution level classifications for the single-factor pollution index (P_i_) and the Nemerow pollution index (P_n_) are shown in Table 1 [28, 29].

**Table 1:** Classification of pollution levels for Pi and Pn.

P_i_	Pollution level	P_n_	Pollution degree
**P**_**i**_ **<1**	Unpolluted	**P≤0.7**	Safe
**1≤P**_**i**_ **<2**	Slightly polluted	**0.7<P≤1**	Precaution
**2≤P**_**i**_ **<3**	Moderately Polluted	**1<P≤2**	Slight pollution
**3≤P**_**i**_ **<5**	Highly polluted	**2<P≤3**	Moderate pollution
**P**_**i**_ **≥5**	Very highly polluted	**P>3**	Heavy pollution

#### Health risk assessment

Surface water heavy metal elements can enter the human body through daily drinking water intake, skin absorption and respiration. For humans, intake and skin absorption are the two main exposure pathways for aquatic heavy metals [30, 31]. The daily dose of water intake and skin absorption, and the carcinogenic risk and non-carcinogenic risk of heavy metals to the human body were determined according to relevant documents from the US EPA [32], using the following equations:

$${\text{ADD}}_{\text{ingestion}}=\frac{{\text{C}}_{\text{i}}\times \text{IR}\times \text{EF}\times \text{ED}}{\text{BW}\times \text{AT}}$$ (3)

$${\text{ADD}}_{\text{dermal}}=\frac{{\text{C}}_{\text{i}}\times \text{SA}\times {\text{K}}_{\text{p}}\times \text{ET}\times \text{EF}\times \text{ED}}{\text{BW}\times \text{AT}}\times 10^{-3}$$ (4)

$$\text{HI}=\sum \text{HQ}=\sum \frac{\text{ADD}}{\text{RfD}}$$ (5)

TCR=∑CR=∑ADD×CSF (6)

Among them: ADD_ingestion_ and ADD_dermal_ are the daily doses for drinking water intake and skin absorption, respectively; HQ is the risk quotient; HI is the risk index; CR is the carcinogenic risk; TCR is the total carcinogenic risk of all exposed metals; C_i_ is the concentration of heavy metals in the water body; IR is the average daily drinking water intake; EF is the exposure frequency; ED is the exposure time; BW is the average body weight; AT is the average exposure time; SA is the skin exposure area; SL is the skin adhesion factor; ET is the exposure time; and K_p_ is the permeability coefficient of heavy metals in water. The specific exposure parameters are shown in Table 2 [33]. Table 3 shows the RfD, CSF and K_p_ values of the heavy metals [34]. HQ and HI are used to describe the non-carcinogenic risk of heavy metals. When HQ or HI<1, there is no non-carcinogenic health risk; otherwise, there is a potential non-carcinogenic health risk, with larger values representing higher risk. CR and TCR are used to describe the carcinogenic risk of heavy metals. When CR<10^−6^, there is no carcinogenic risk; when CR is between 10^−6^–10^−4^, the risk is acceptable; and when CR>10^−4^, the heavy metals in the water body are likely to cause cancer risk to the human body.

**Table 2:** Exposure parameters for the health risk assessment models.

Parameters	Unit	Value	
		Child	Adult
**IR**	L·d^-1^	0.64	2
**EF**	d·year^-1^	350	350
**ED**	years	6	30
**BW**	kg	15	70
**AT**	d	2190 (For non-carcinogens)	10950 (For non-carcinogens)
		25550 (For carcinogens)	25550 (For carcinogens)
**SA**	cm^2^	6600	18000
**ET**	h	1	0.58

**Table 3:** RfD, CSF and Kp of heavy metals.

Element	RfD_ingestion_	RfD_dermal_	CSF_ingestion_	CSF_dermal_	K_p_(cm/h)
	(μg/kg/day)	(μg/kg/day)	(mg/kg/day)^-1^	(mg/kg/day)^-1^	
**Cu**	40	8			0.001
**Pb**	1.4	0.42			0.0001
**Zn**	300	60			0.0006
**As**	0.3	0.285	1.5	3.66	0.001
**Mn**	24	0.96			0.001
**Cd**	0.5	0.025			0.001
**Cr**	3	0.075			0.002
**Ni**	20	0.8			0.0002
**Hg**	0.3	0.021			0.001

### Statistical analysis

Correlation analysis, cluster analysis and principal component analysis (PCA) can effectively reflect the source of heavy metals [35, 36]. In order to understand the heavy metal sources in the Rongna River, this study used IBM SPSS Statistics 24 software to conduct Pearson correlation analysis (two-tailed), cluster analysis and PCA on the heavy metals and pH in the water at the sampling points. For the PCA, the principal component was calculated based on the correlation matrix, VARIMAX was used to normalize the rotation, and the principal component was extracted only when the eigenvalue was greater than or equal to 1.

## Results and discussion

### Distribution characteristics of heavy metals in the water of Rongna River

The heavy metal content and pH characteristics of the Rongna River water are shown in Table 4. The pH of the water body ranged from 6.52 to 8.46, with an average value of 7.26, which meets the corresponding Grade III national surface water standard [26]. The pH range of AMD was 2.86–3.06, with an average value of 2.98, which is much lower than the corresponding Grade III national surface water standard and denotes serious acidification. Under the action of humans, as well as some natural destructive forces, the original stable protective layer on the metal sulfide deposits and surrounding rocks can be destroyed, which exposes them to the atmospheric oxygen-containing environment, resulting in a large amount of AMD [6]. Acidic water increases the solubility of heavy metals, which further increases their diffusion capacity [17]. Therefore, the acidic water produced in the mining area may be the main reason that the Rongna River is polluted by heavy metals.

**Table 4:** Characteristics of heavy metal concentrations in the water.

Parameters	Rongna river		AMD		Bolong river		Grade III
	Range	Mean	Range	Mean	Range	Mean	
**Cu/(μg/L)**	1.89–806.00	280.98±259.04	1890.00–2272.00	2072.67±156.4	5.42–7.37	6.50±0.81	1000
**Pb/(μg/L)**	0.49–2.41	1.32±0.63	1.21–2.17	1.65±0.4	0.34–0.64	0.52±0.13	50
**Zn/(μg/L)**	13.00–415.00	178.66±135.39	1321.00–1515.00	1404.67±81.41	39.80–56.70	46.73±7.23	1000
**As/(μg/L)**	0.01–3.83	0.88±1.27	0.07–0.64	0.29±0.25	1.09–4.96	2.43±1.79	50
**Mn/(μg/L)**	43.10–2041.00	867.37±678.69	7488.00–8072.00	7835±250.78	37.80–51.90	46.47±6.19	100
**Cd/(μg/L)**	0.12–0.64	0.43±0.16	0.84–1.54	1.2±0.29	0.11–0.54	0.35±0.18	5
**Cr/(μg/L)**	1.56–6.37	4.24±1.58	3.13–7.24	4.87±1.73	1.74–3.08	2.32±0.56	50
**Ni/(μg/L)**	7.45–60.10	28.44±16.8	167.00–189.00	178.67±9.03	10.7–18.22	13.84±3.19	20
**Hg/(μg/L)**	0.001–0.012	0.0053±0.0042	0.001–0.005	0.0023±0.0019	0.010–0.011	0.0097±0.0012	0.1
**pH**	6.52–8.46	7.26±0.67	2.86–3.06	2.98±0.08	7.95–8.06	7.95±0.09	6–9

**Fig 3:**
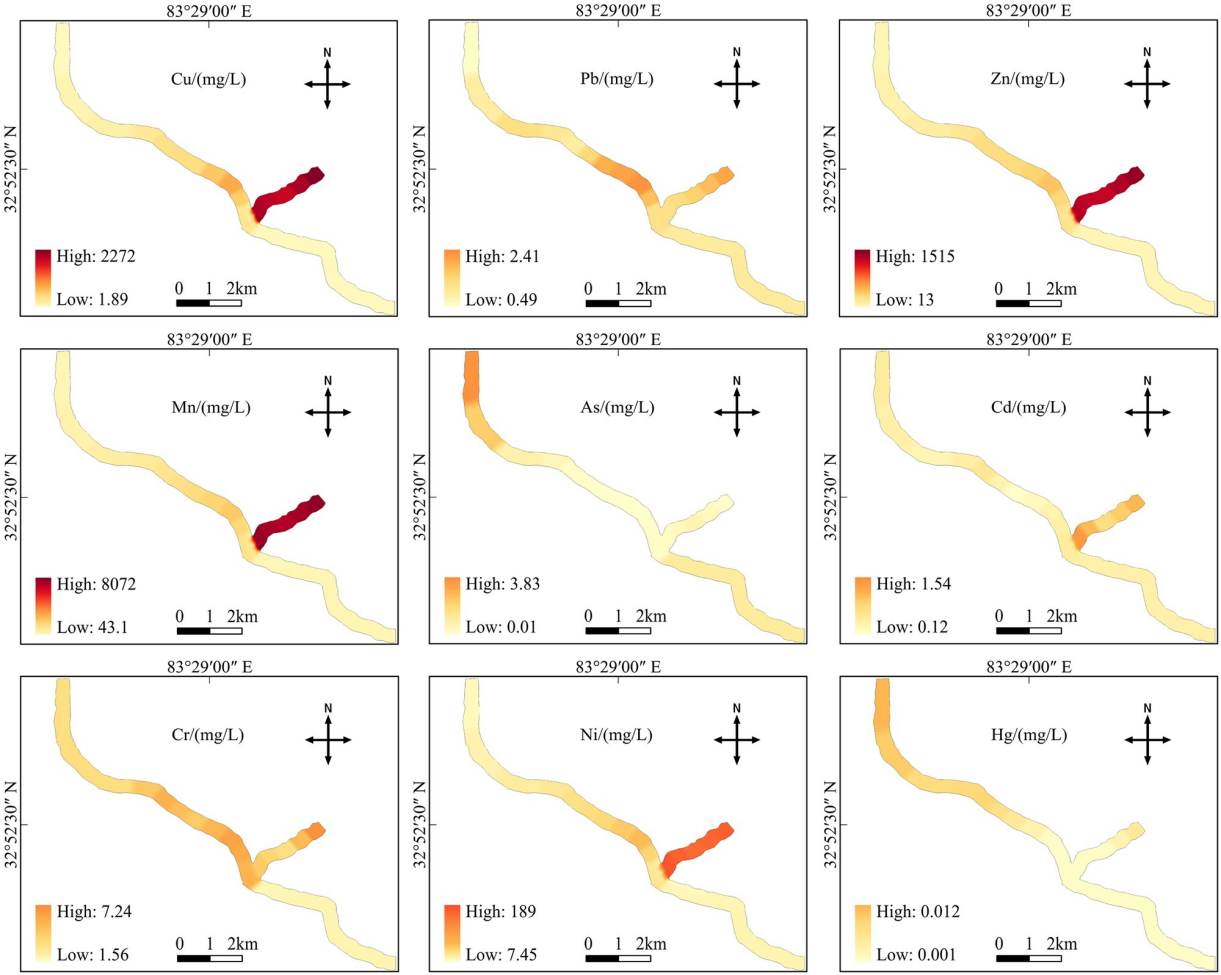
Spatial distribution of heavy metals in the Rongna River water. The location coordinate map of the study area was drawn according to the USGS National Map Viewer.

Concentrations of the heavy metals Cu, Pb, Zn, As, Cd, Cr, Ni and Hg in the water of Rongna River were all within the limits of the Grade III national surface water environmental quality standard, whereas both Mn and Ni exceeded the Grade III standard by 8.67 and 1.42 times, respectively. The concentrations of Cu, Zn, Mn and Ni in the AMD exceed the Grade III standard by 2.07, 1.40, 78.35 and 8.93 times, respectively. In the river sections before and after the AMD inflow point, the spatial distribution of heavy metal concentrations in the Rongna River changed substantially (Fig 3). The concentration of heavy metals in the upper reaches of the Rongna River (R1) was similar to that of the Bolong River, which was used as the control because it is far away from the mining area. This shows that without the influx of AMD, the natural weathering of rocks may not cause serious heavy metal pollution to the river. The heavy metal concentrations for Cu, Zn, Mn and Ni in the AMD were 318.71, 30.06, 168.62 and 12.91 times higher, respectively, than those in the Bolong River. After the AMD entered the Rongna River (R2-R6), the heavy metals in the water were significantly greater than the Bolong River. The concentrations of Cu, Zn, Mn, Pb, Cr and Ni in the water from sites R2-R6 were 60.36, 5.23, 25.65, 3.00, 2.15 and 2.60 times higher, respectively, than those in the Bolong River. The Cu, Zn, and Mn concentrations in the polluted reaches of the Rongna River were greater larger than those of the Heihe River (Table 5), which is distributed in the mining area but has no AMD discharge [37]; Conversely, the content of heavy metals in Rongna River was less than that in the Gyamaxung-chu River, which is distributed in the mining area and polluted by AMD [38]. Comparing the rivers in two different mining areas shows that AMD is the main factor causing pollution of the rivers in the mining area. At the end of the Rongna River (R7), the heavy metal concentrations in the water were close to those in the Bolong River. Furthermore, compared with the source of some rivers in northern Tibet and the Lhasa River distributed around a city, the concentration of heavy metals at the end of the Rongna River was close to that of the Yellow River, Buha River, Shule River and Lhasa River [39, 40]. This indicates that after the long-distance self-purification of the river, the heavy metal concentrations in the river returned to normal levels.

**Table 5:** Comparison of heavy metal contents in other rivers in Tibet.

Location	Cu	Pb	Zn	As	Mn	Cd	Cr	Ni	Hg	pH
**Yellow River**	1	0.1	4.4	1.2	3.3	<0.007	1.8	—	—	8.0–8.6
**Buha He**	1.4	<0.05	3.7	0.9	3.8	N.D.	2	—	—	8.5
**Shule He**	0.8	0.1	1.5	1.4	6.3	<0.004	2	—	—	8.3–8.8
**Lhasa River**	2.863	0.056	0.829	3.071	6.237	0.042	3.156	—	0.005	8.8
**Heihe River**	6.02	6.14	66.70	2.68	46.75	0.65	6.57	20.37	—	8.6–8.7
**Gyamaxung-chu**	5800	695.67	2454.67	—	1061	2.87	2.30	23.20	—	—

Note: The heavy metal units in the table are all μg/L.

### Environmental risk assessment of heavy metals

#### Single-factor evaluation method and Nemerow pollution index

**Fig 4:**
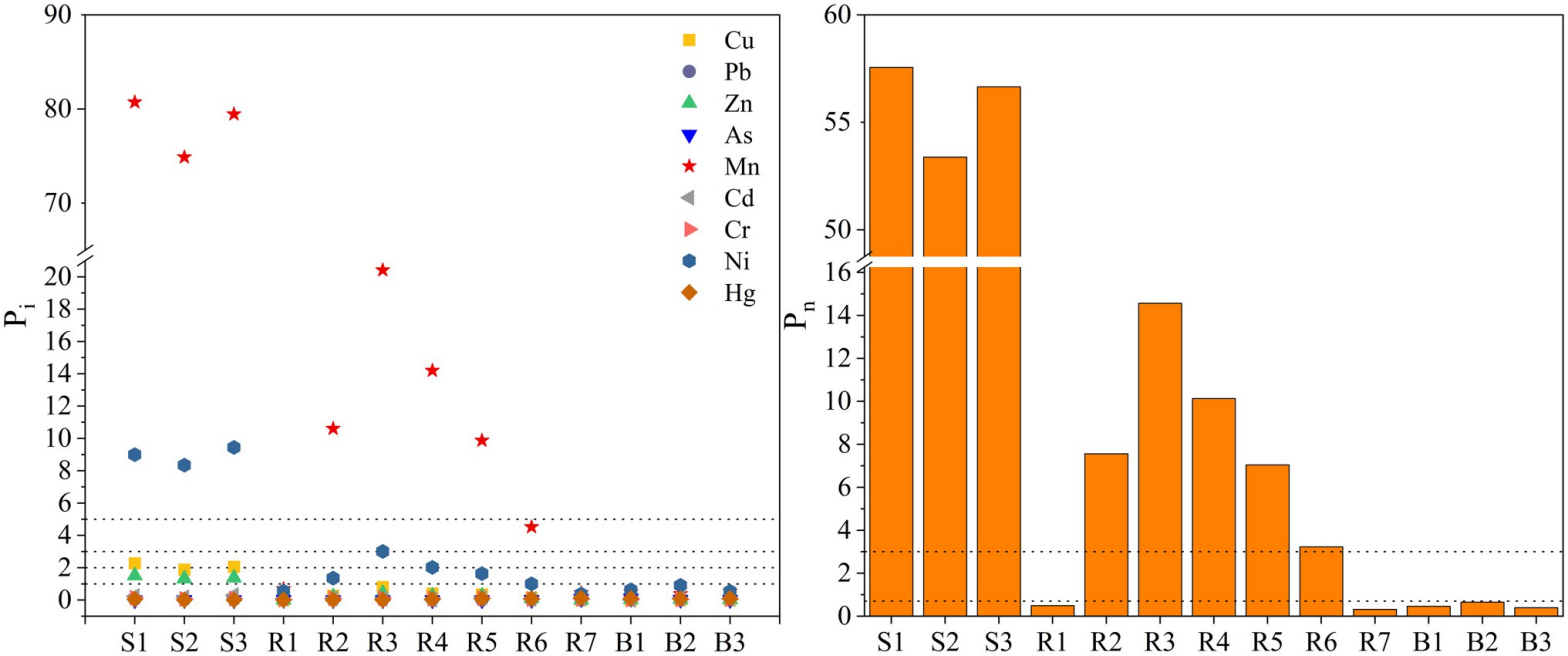
Pi and Pn of heavy metals in the Rongna River.

Fig 4 shows the results of the single-factor pollution index (P_i_) and Nemerow pollution index (P_n_) at different points along the Rongna River. The P_i_ of all heavy metals in the unpolluted section of R1, the upstream section of the Rongna River, was less than 1, which represents no pollution. In the AMD (S1-S3), the single-factor pollution index of Mn and Ni was greater than 5, which represents extremely serious pollution, while the average P_i_ value of Cu was 2.07 (moderate pollution). The average P_i_ of Zn was 1.40 (slight pollution), while the P_i_ of other heavy metals was less than 1 (no pollution). Where AMD flows into the river section (R2-R6), the average P_i_ of Mn was 11.92 (extremely serious pollution), and the average P_i_ of Ni was 1.81 (light pollution). The P_i_ of all heavy metals at the end of the Rongna River (R7) was less than 1 (no pollution). The characteristics of P_i_ correspond to the spatial distribution characteristics of heavy metals in the Rongna River that were presented in the previous section (3.1). This indicates that the heavy metal pollution in the Rongna River water body is mainly affected by deposit-associated AMD.

The P_n_ in both the AMD (S1-S3) and the main reach of Rongna River (R2-R6) were greater than 3, indicating heavy pollution. This shows that AMD caused most of the Rongna River to be polluted. The upper (R1) and lower (R2) reaches of Rongna River and the Bolong River (control) were all less than 0.7, which indicates a clean state. The P_n_ of the R2-R6 reach showed a trend of first increasing and then decreasing as the distance of the river increased. This indicates that the heavy metals are gradually diluted by the clean river water and, along with self-purification of the river itself, the water basically returns to an unpolluted state by the time it reaches the end of the river.

#### Health risk assessment

**Fig 5:**
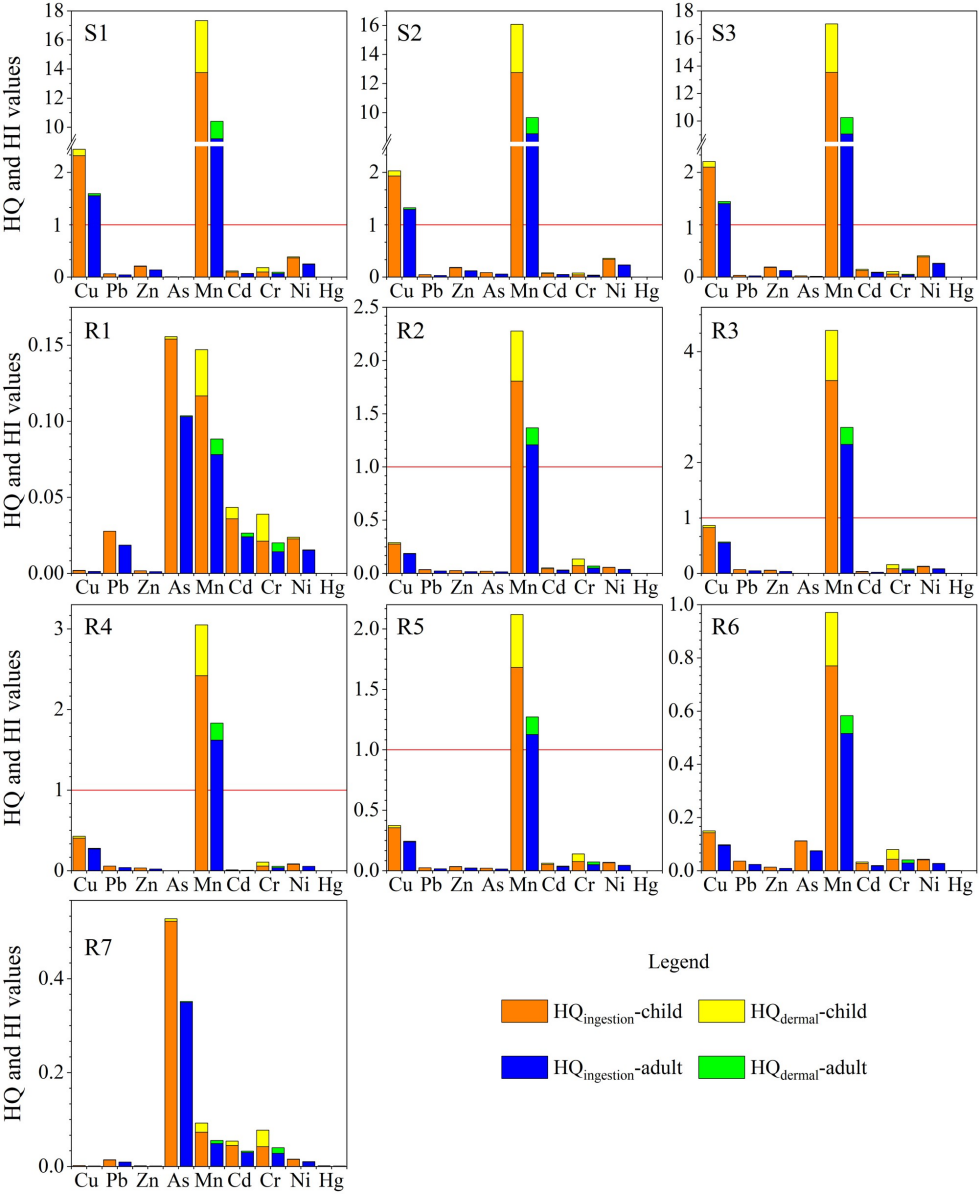
HQ and HI of the Rongna River water.

Tables 6 and 7 show the calculated non-carcinogenic and carcinogenic risk coefficients HQ and CR for children and adults based on the model provided by the US EPA. These values can be used to judge the potential carcinogenic and non-carcinogenic risks of polluted water to the exposed population. It can be seen from Fig 5 that in the AMD (S1-S3), the HQ_ingestion_, HQ_dermal_ and HI values of the element Mn for children and adults are all greater than 1, indicating that ingestion or skin exposure to Mn will cause a certain non-carcinogenic risk to humans. The HQ_ingestion_ and HI values of Cu for children and adults were greater than 1, while HQ_dermal_ was less than 1, indicating that water intake of Cu will cause potential non-carcinogenic health risks to children and adults, whereas skin contact with Cu will not cause potential non-carcinogenic risks. At sites R2-R5, the HQ_ingestion_ and HI values of Mn to children and adults were greater than 1, while HQ_dermal_ was less than 1, indicating that oral ingestion of Mn poses a potential non-carcinogenic risk to children and adults, while skin contact does not cause potential non-carcinogenic risks. At sites R1, R6 and R7, the HI for adults and children was less than 1, indicating that exposure at these sites would not pose a potential non-carcinogenic risk to children and adults. The HQ_ingestion_ of all heavy metal elements in the river water was greater than HQ_dermal_. This shows that oral intake is the main exposure mode for heavy metal non-carcinogenic risk, which agrees with the results of a previous study [41]. For both HQ and HI, the values for children were significantly higher than those for adults, indicating that children have a higher non-carcinogenic risk under the same environmental conditions. Previous studies have also come to this conclusion [30, 31].

**Table 6:** Hazard quotient (HQ) and cancer risk (CR) of heavy metals from Rongna River for children.

**Site**	**Cu**	**Pb**	**Zn**	**As**	**Mn**	**Cd**	**Cr**	**Ni**	**Hg**	**As**
	**HQ_ingestion_**									**CR_ingestion_**
**S1**	2.32E+00	6.34E-02	2.07E-01	9.55E-03	1.38E+01	9.90E-02	9.87E-02	3.68E-01	6.82E-04	3.68E-07
**S2**	1.93E+00	4.59E-02	1.80E-01	8.73E-02	1.28E+01	6.87E-02	4.27E-02	3.42E-01	1.36E-04	3.37E-06
**S3**	2.10E+00	3.54E-02	1.88E-01	2.18E-02	1.35E+01	1.26E-01	5.80E-02	3.87E-01	1.36E-04	8.42E-07
**R1**	1.93E-03	2.78E-02	1.77E-03	1.54E-01	1.17E-01	3.60E-02	2.13E-02	2.27E-02	1.36E-04	5.94E-06
**R2**	2.76E-01	3.59E-02	2.70E-02	2.18E-02	1.81E+00	4.42E-02	7.45E-02	5.56E-02	1.36E-04	8.42E-07
**R3**	8.24E-01	7.04E-02	5.66E-02	4.09E-03	3.48E+00	3.11E-02	8.69E-02	1.23E-01	1.36E-04	1.58E-07
**R4**	4.07E-01	5.96E-02	3.50E-02	1.36E-03	2.42E+00	9.82E-03	5.92E-02	8.26E-02	6.82E-04	5.26E-08
**R5**	3.57E-01	2.54E-02	3.44E-02	2.18E-02	1.68E+00	5.24E-02	7.69E-02	6.69E-02	1.23E-03	8.42E-07
**R6**	1.43E-01	3.65E-02	1.39E-02	1.12E-01	7.71E-01	2.78E-02	4.38E-02	4.11E-02	1.09E-03	4.31E-06
**R7**	1.99E-03	1.43E-02	1.85E-03	5.22E-01	7.35E-02	4.50E-02	4.24E-02	1.52E-02	1.64E-03	2.01E-05
	**HQ**_**dermal**_									**CR**_**dermal**_
**S1**	1.21E-01	2.20E-04	6.45E-03	1.05E-04	3.58E+00	2.06E-02	8.22E-02	1.92E-02	1.01E-04	9.35E-09
**S2**	1.01E-01	1.59E-04	5.62E-03	9.56E-04	3.32E+00	1.43E-02	3.55E-02	1.78E-02	2.03E-05	8.55E-08
**S3**	1.09E-01	1.23E-04	5.87E-03	2.39E-04	3.52E+00	2.62E-02	4.83E-02	2.01E-02	2.03E-05	2.14E-08
**R1**	1.01E-04	9.63E-05	5.53E-05	1.69E-03	3.04E-02	7.49E-03	1.77E-02	1.18E-03	2.03E-05	1.51E-07
**R2**	1.44E-02	1.25E-04	8.43E-04	2.39E-04	4.70E-01	9.20E-03	6.20E-02	2.90E-03	2.03E-05	2.14E-08
**R3**	4.29E-02	2.44E-04	1.77E-03	4.48E-05	9.05E-01	6.47E-03	7.23E-02	6.40E-03	2.03E-05	4.01E-09
**R4**	2.12E-02	2.07E-04	1.09E-03	1.49E-05	6.30E-01	2.04E-03	4.93E-02	4.30E-03	1.01E-04	1.34E-09
**R5**	1.86E-02	8.82E-05	1.07E-03	2.39E-04	4.38E-01	1.09E-02	6.40E-02	3.48E-03	1.82E-04	2.14E-08
**R6**	7.45E-03	1.27E-04	4.34E-04	1.22E-03	2.00E-01	5.79E-03	3.64E-02	2.14E-03	1.62E-04	1.10E-07
**R7**	1.04E-04	4.97E-05	5.79E-05	5.72E-03	1.91E-02	9.37E-03	3.53E-02	7.93E-04	2.43E-04	5.12E-07

**Table 7:** Hazard quotient (HQ) and cancer risk (CR) of heavy metals from Rongna River for adults.

**Site**	**Cu**	**Pb**	**Zn**	**As**	**Mn**	**Cd**	**Cr**	**Ni**	**Hg**	**As**
	**HQ**_**ingestion**_									**CR**_**ingestion**_
**S1**	1.56E+00	4.25E-02	1.38E-01	6.39E-03	9.21E+00	6.63E-02	6.61E-02	2.47E-01	4.57E-04	1.23E-06
**S2**	1.29E+00	3.07E-02	1.21E-01	5.84E-02	8.55E+00	4.60E-02	2.86E-02	2.29E-01	9.13E-05	1.13E-05
**S3**	1.41E+00	2.37E-02	1.26E-01	1.46E-02	9.07E+00	8.44E-02	3.88E-02	2.59E-01	9.13E-05	2.82E-06
**R1**	1.29E-03	1.86E-02	1.19E-03	1.03E-01	7.82E-02	2.41E-02	1.42E-02	1.52E-02	9.13E-05	1.99E-05
**R2**	1.85E-01	2.41E-02	1.81E-02	1.46E-02	1.21E+00	2.96E-02	4.99E-02	3.73E-02	9.13E-05	2.82E-06
**R3**	5.52E-01	4.72E-02	3.79E-02	2.74E-03	2.33E+00	2.08E-02	5.82E-02	8.23E-02	9.13E-05	5.28E-07
**R4**	2.73E-01	3.99E-02	2.35E-02	9.13E-04	1.62E+00	6.58E-03	3.96E-02	5.53E-02	4.57E-04	1.76E-07
**R5**	2.39E-01	1.70E-02	2.30E-02	1.46E-02	1.13E+00	3.51E-02	5.15E-02	4.48E-02	8.22E-04	2.82E-06
**R6**	9.59E-02	2.45E-02	9.32E-03	7.49E-02	5.16E-01	1.86E-02	2.93E-02	2.75E-02	7.31E-04	1.44E-05
**R7**	1.34E-03	9.59E-03	1.24E-03	3.50E-01	4.92E-02	3.01E-02	2.84E-02	1.02E-02	1.10E-03	6.75E-05
	**HQ**_**dermal**_									**CR**_**dermal**_
**S1**	4.06E-02	7.39E-05	2.17E-03	3.51E-05	1.20E+00	6.92E-03	2.76E-02	6.44E-03	3.41E-05	1.57E-08
**S2**	3.38E-02	5.35E-05	1.89E-03	3.21E-04	1.12E+00	4.81E-03	1.19E-02	5.97E-03	6.81E-06	1.44E-07
**S3**	3.68E-02	4.12E-05	1.97E-03	8.03E-05	1.18E+00	8.81E-03	1.62E-02	6.76E-03	6.81E-06	3.59E-08
**R1**	3.38E-05	3.23E-05	1.86E-05	5.67E-04	1.02E-02	2.52E-03	5.95E-03	3.97E-04	6.81E-06	2.53E-07
**R2**	4.83E-03	4.19E-05	2.83E-04	8.03E-05	1.58E-01	3.09E-03	2.08E-02	9.72E-04	6.81E-06	3.59E-08
**R3**	1.44E-02	8.21E-05	5.94E-04	1.51E-05	3.04E-01	2.17E-03	2.43E-02	2.15E-03	6.81E-06	6.73E-09
**R4**	7.11E-03	6.95E-05	3.68E-04	5.02E-06	2.12E-01	6.86E-04	1.66E-02	1.44E-03	3.41E-05	2.24E-09
**R5**	6.24E-03	2.96E-05	3.60E-04	8.03E-05	1.47E-01	3.66E-03	2.15E-02	1.17E-03	6.13E-05	3.59E-08
**R6**	2.50E-03	4.26E-05	1.46E-04	4.11E-04	6.73E-02	1.94E-03	1.22E-02	7.19E-04	5.45E-05	1.84E-07
**R7**	3.49E-05	1.67E-05	1.94E-05	1.92E-03	6.42E-03	3.15E-03	1.19E-02	2.66E-04	8.17E-05	8.59E-07

**Fig 6:**
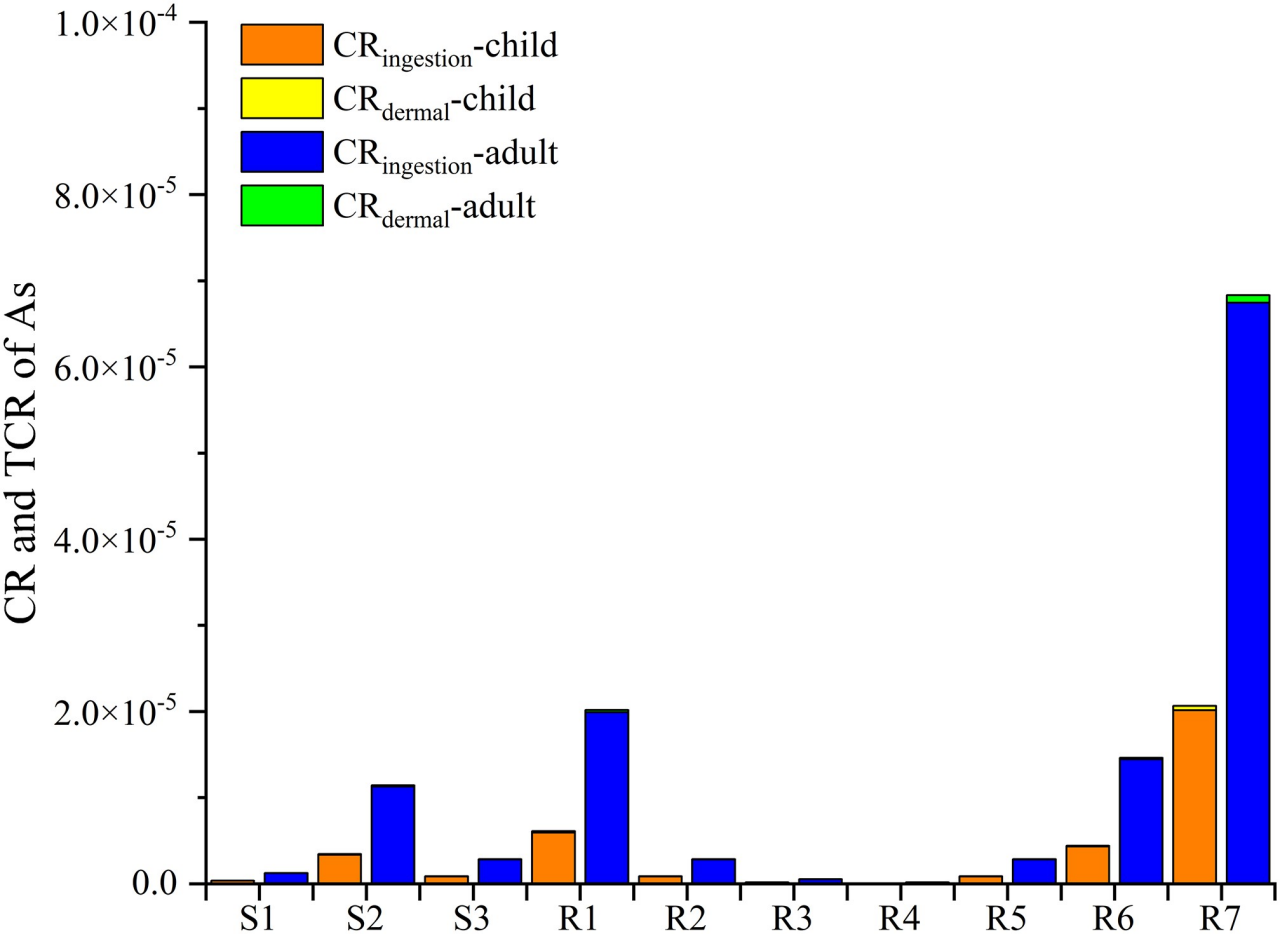
CR and TCR of As in the Rongna River water.

The US EPA classifies As a carcinogen that is harmful to humans. This study calculated the carcinogenic risk of As to children and adults using Eqs (3)–(6). It can be seen from the results (Fig 6) that the carcinogenic risk through ingestion and skin exposure was between 1.34×10^−9^–6.75×10^−5^, which is less than 10^−4^, the acceptable carcinogenic risk stated by the US EPA. At the same time, the carcinogenic risk for adults via the oral intake and skin exposure routes was higher than that of children, which may be attributed to the larger amount of water consumed by adults and their larger skin area.

### The impact of heavy metal pollution on the distribution of phytoplankton

As the most important phytoplankton in aquatic ecosystems, algae are widely distributed in rivers, lakes and seas [42]. Algae are not only closely related to their living environment, but they also play an important role in processes related to material circulation, energy conversion and information transmission. Different types of algae have different sensitivities to changes in the aquatic environment, and their species composition, community structure and biomass are closely related to the environment factors in which they live [43, 44]. Moreover, they can be used as indicators of water quality changes and a means to monitor and evaluate the health of the aquatic environment because some algae have a fast growth cycle and the community structure is very sensitive to environmental changes [45].

Table 8 shows the distribution characteristics of algae at the different water sites. A total of 30 algal species were detected in the study area, namely 5 species of Cyanophyta, 23 species of Diatoms and 2 species of Chlorophyta. Among these 30 phytoplankton species, *Pseudoanabaena* sp. exhibited the highest average density in the entire river at 20.14×10^4^ cell/L and a maximum dominance of 0.645. *Melosira* sp. (3.068×10^4^ cell/L and 0.098 dominance) and *Pinnularia* sp. (2.044×10^4^ cell/L and 0.065 dominance) were the next most predominant. The density of phytoplankton in the AMD was 0.1×10^4^ cell/L. The density of photoplankton increased from R1 to R6, with R6 registering 110.85×10^4^ cell/L.

**Table 8:** Distribution characteristics of photoplankton in the study area (×10^4^ cell/L).

Category	species	R1	R2	R3	R4	R5	R6	R7	S1-S3
**Cyanophyta**	*Chroococcus* sp.								0.1
	*Pseudoanabaena* sp.				19.4	55.6	86.15		
	*Oscillatoria* sp.				4.4		5.15		
	*Anabaena* sp.				1.7				
	*Aphanizomenon* sp.				4	8.25			
**Diatoms**	Melosira sp.			10.35	1.8	1.55	10.3	0.55	
	*Tabellaria* sp.				0.15	0.4	0.1	0.05	
	*Diatoma vulgare*	0.1					0.1	0.05	
	*Fragilaria* sp.			1.15	0.55	0.7	0.6		
	*Fragilaria intermedia*			0.35	0.15	0.55	0.6		
	*Synedra* sp.		0.2	0.05					
	*Eunotia* sp.	0.05		0.15					
	Navicula sp.	0.4	1.4	1	1.15	2.4	1.9	0.8	
	*Navicula bacilloides Hust*	0.05							
	*Pinnularia* sp.	0.4	1.45	1.7	2.8	4	4.7	1.3	
	*Pinnularia viridis*		0.1						
	*Cymbella* sp.	0.1	0.8	0.45	0.5	0.8	0.8	0.2	
	*Cymbella aspera*			0.05					
	*Cymbella cistula*	0.1	0.5	0.45	0.1	0.15		0.15	
	*Cymbella turgidula*			0.05					
	*Gomphonema* sp.	0.05		0.05	0.05		0.1	0.1	
	*Gomphonema constrictum*					0.05			
	*Achnanthes* sp.		0.05						
	*Hantzschia* sp.					0.05			
	*Nitzschia* sp.	0.1	0.5	0.15	0.55	0.55	0.2	0.15	
	*Nitzschia linearis*		0.05						
	*Cymatopleura* sp.						0.15		
	*Surirella* sp.	0.05	0.05	0.05					
**Chlorophyta**	*Crucigenia quadrata*		0.05	0.1	0.05				
	*Ulothrix* sp.	0.55							

Due to the different water pollution levels at the different sites of the Rongna River, the corresponding phytoplankton species composition and population abundance varied greatly. A total of 11 algal species were found in the upstream clean river section (R1), specifically 10 species of Diatoma and 1 species of Chlorophyta. *Navicula bacilloides Hust* and *Ulothrix* sp. were only found in the clean river water in the upper reaches of the river. *Diatoma vulgare* appeared in both the upper and lower reaches of the Rongna River. Therefore, these three types of algae can be used as indicator species for clean water bodies.The diversity of algae was low in the AMD, due to the existence of a high concentration of heavy metal elements, especially Cu, which is toxic to algae [46]. In fact, only *Chroococcus* sp. was observed in the AMD. This indicates that *Chroococcus* sp. can survive in AMD, has strong adaptability to acid and heavy metal pollution, and can be used as a typical indicator alga for AMD. Algae at sites R2-R6 included *Pseudoanabaena* sp., *Melosira* sp., *Aphanizomenon* sp., *Anabaena* sp., *Pinnularia* sp., *Navicula* sp., *Navicula* sp., *Oscillatoria* sp. and *Cymbella* sp. At the end of the Rongna River (R7), the dominant community members were *Plumbonia*, *Navicula* and *Melosira*, with dominance of 0.388, 0.239 and 0.164, respectively. The comparison found that cyanobacteria only appeared in the AMD and polluted sections of the Rongna River, indicating that cyanobacteria can adapt to heavy metal-polluted environments and can be used as an indicator of heavy metal pollution in river water. This is related to the strong tolerance of cyanobacteria to heavy metals [47]. Pearson correlation coefficients between the density of plankton and heavy metal concentrations are shown in Table 9. It can be seen that the pollutant elements Cu, Zn, Mn, Cd, and Ni are negatively correlated with phytoplankton biomass, indicating that the concentration of these heavy metals exceeds the tolerance range of algae and affects their normal growth. The cyanobacteria and Hg were positively correlated at the level of 0.05, which is similar to previous studies, indicating that Hg has a certain promoting effect on the growth of algae within a safe concentration range [48]. Chlorophyta were negatively correlated with Hg concentrations, which may be because the detected Chlorophyta are more sensitive to Hg.

**Table 9:** Pearson correlation coefficients between the density of photoplankton and heavy metal concentration.

Category	Cu	Pb	Zn	As	Mn	Cd	Cr	Ni	Hg
**Cyanophyta**	-0.535	-0.633	-0.497	0.786	-0.512	-0.327	-0.756	-0.508	0.853*
**Diatoms**	-0.630	-0.187	-0.673	0.616	-0.687	-0.635	-0.605	-0.651	0.240
**Chlorophyta**	-0.166	0.567	-0.251	-0.482	-0.239	-0.480	0.176	-0.213	-.823*

* Correlation is significant at the 0.05 level (2-tailed).

### Source analysis of heavy metals

**Fig 7:**
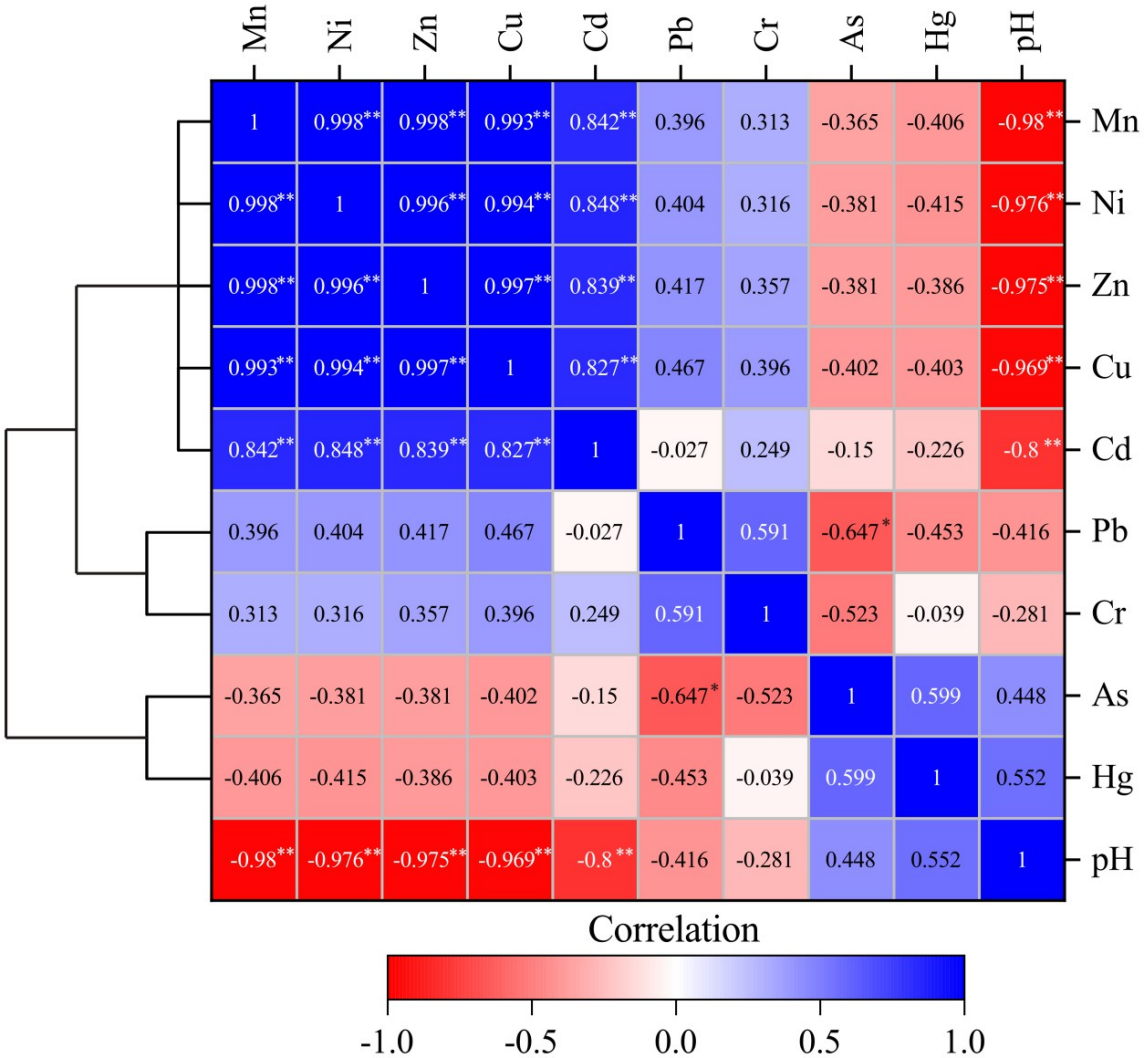
Heatmap of Pearson’s correlation coefficients combined with cluster analysis of heavy metals and pH. ** Correlation is significant at the 0.01 level. * Correlation is significant at the 0.05 level.

Correlation analysis is an important basis for determining the source of heavy metal elements. A significant correlation between the metal elements means that the elements are homologous or have some relevance [49]. Correlation analysis of the contents of 9 heavy metal elements in the water at each sampling point (Fig 7) showed that Cu, Zn, Ni, Mn and Cd exhibited extremely significant positive correlations (P<0.01), indicating that Cu, Zn, Ni, Mn and Cd have homology. The pH and the heavy metals Cu, Zn, Ni, Mn and Cd showed a very significant negative correlation (P<0.01). The lower the pH, the higher the content of heavy metals in the water, indicating that the heavy metals Cu, Zn, Ni, Mn, Cd and Mn in the water of the Rongna River are related to AMD. AMD increases the solubility of heavy metals, causing a large amount of heavy metal ions to enter the river with the AMD. Cluster analysis is used to group heavy metals with homologous characteristics to determine their source. In the water of the Ronna River, three distinct clusters were identified (Fig 7). Mn, Ni, Zn, Cu and Cd, which had higher content, were classified into the same cluster, and these elements may come from AMD. The content of Pb and Cr were slightly higher than that of the Bolong River, and they were grouped into the same cluster. The source of these two heavy metals may be related to the oxidation of sulfide mines. The contents of As and Hg were similar to those of the Bolong River and were classified into the same cluster, which indicates that these two heavy metals may originate from lithogenic sources.

**Fig 8:**
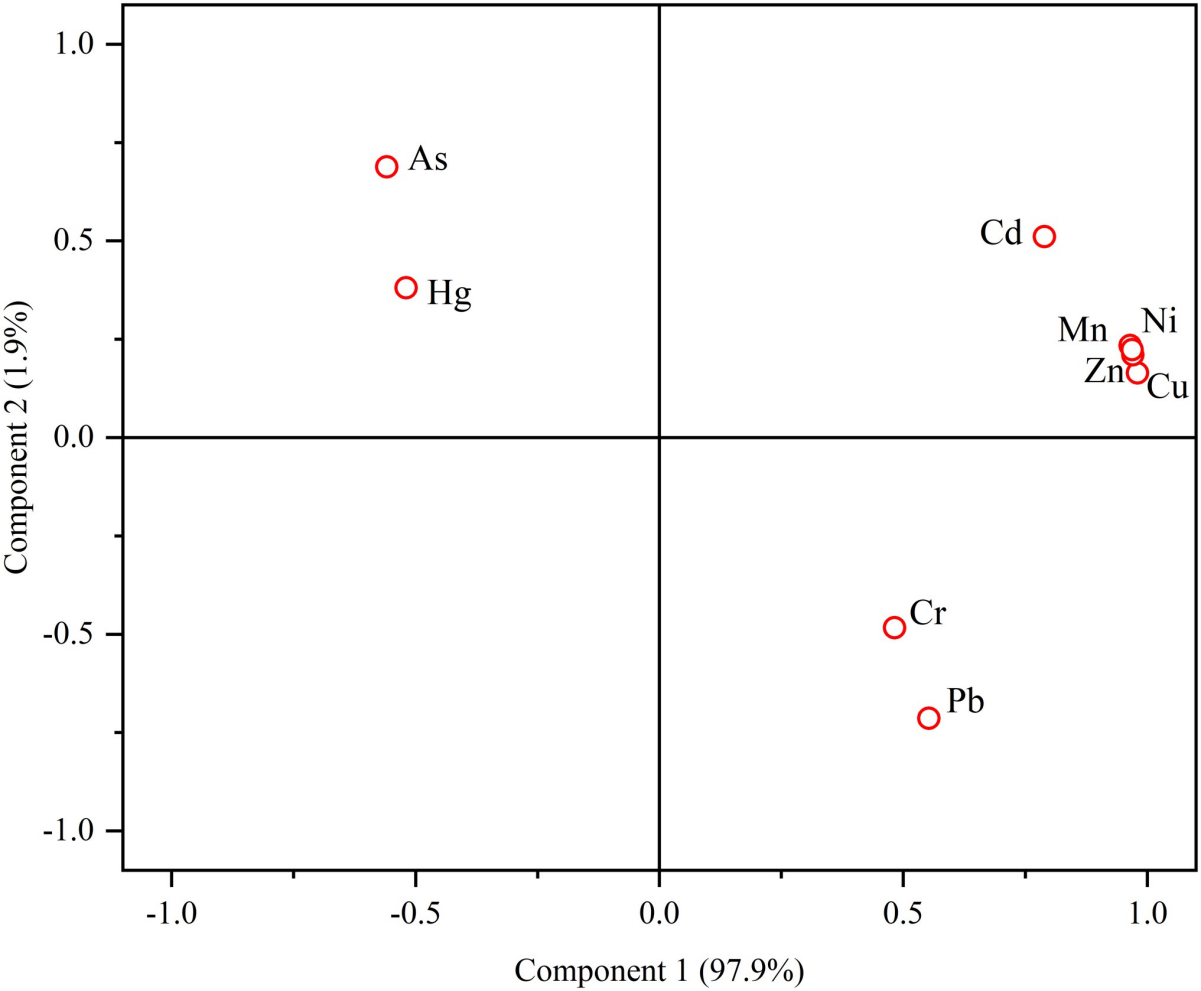
Score plot for the principal component analysis of heavy metals in the Rongna River.

PCA can effectively determine the source of heavy metal pollution [50]. Table 10 shows the PCA results for the heavy metals. Two principal components with a rotation value greater than 1 were extracted, and the cumulative contribution rate of the two principal components reached 81.27%, which can explain most of the information for the heavy metal elements. The principal component loading diagram (Fig 8) shows that the contribution rate of principal component 1 reached 61.30%, and mainly represented Cu, Zn, Ni, Mn and Cd. This agrees with the result of the correlation analysis and cluster analysis, further suggesting that Cu, Zn, Ni, Mn and Cd may come from the same source. Moreover, the main pollution in the water was also Cu, Zn, Ni, Mn and Cd, indicating that these heavy metals are mainly derived from AMD. Principal component 2 explained 1.9% of the total variance. The loadings of Hg and As were 69% and 51%, respectively, and the concentrations of Hg, As, Cr and Pb were similar to those of the control river Bolong River. This indicates that Hg, As, Cr and Pb may come from a background source related to rock weathering in the environment.

**Table 10:** PCA results of heavy metals in the Rongna River.

Component	Initial eigenvalues			Rotation sums of squared loadings			Element	Principal component	
	Total	%of variance	Cumulative%	Total	%of variance	Cumulative%		1	2
**1**	5.52	61.30	61.30	5.52	61.30	61.30	Cu	0.98	0.16
**2**	1.80	19.98	81.27	1.80	19.98	81.27	Pb	0.55	-0.71
**3**	0.98	10.90	92.17				Zn	0.97	0.21
**4**	0.45	4.96	97.13				As	-0.56	0.69
**5**	0.22	2.45	99.58				Mn	0.97	0.23
**6**	0.03	0.39	99.97				Cd	0.79	0.51
**7**	0.002	0.021	99.99				Cr	0.48	-0.48
**8**	0.001	0.012	100.00				Ni	0.97	0.22
**9**	0	0	100.00				Hg	-0.52	0.38

## Conclusions

In this study, the concentration characteristics of heavy metals and the distribution of phytoplankton in water from the Rongna River within the unmined Tiegelongnan deposit area were investigated, and a risk assessment of heavy metal pollution in the river water was carried out. The results show that naturally occurring AMD caused serious heavy metal pollution in the Rongna River. Cu, Zn, Mn and Ni in the AMD exceeded the Grade III national surface water environmental quality standard. After the AMD flows into the river, Mn and Ni exceeded the Grade III national surface water environmental quality standard, while the concentrations of Cu, Zn, Mn, Pb, Cr and Ni were 60.36, 5.23, 25.65, 3.00, 2.15 and 2.60 times higher, respectively, than those in the water of the control river Bolong. The results of the heavy metal pollution evaluation indicate that the Rongna River is heavily polluted by heavy metals, with Mn posing a certain non-carcinogenic risk to humans. Thus, the water is no longer suitable for drinking or bathing. There were 30 species of algae detected in the Rongna River. The phytoplankton biomass was negatively correlated with the concentration of major heavy metals, indicating that the heavy metal concentration exceeded the tolerance limit of phytoplankton and thus affected their normal growth. Statistical analysis shows that the heavy metals Cu, Zn, Ni, Mn and Cd are mainly sourced from AMD, while Hg, As, Cr and Pb may come from rock weathering.
